# Base-resolution DNA methylation landscape of zebrafish brain and liver

**DOI:** 10.1016/j.gdata.2014.10.008

**Published:** 2014-10-16

**Authors:** Aniruddha Chatterjee, Peter A. Stockwell, Julia A. Horsfield, Ian M. Morison, Shinichi Nakagawa

**Affiliations:** a Department of Pathology, Dunedin School of Medicine, University of Otago, 270 Great King Street, Dunedin 9054, New Zealand; b Gravida: National Centre for Growth and Development, 85 Park Road, Grafton, Auckland 1142, New Zealand; c Department of Biochemistry, University of Otago, 710 Cumberland Street, Dunedin 9054, New Zealand; d Otago Genomics and Bioinformatics Facility, Centre for Innovation, 87 St David Street, Dunedin 9016, New Zealand; e Department of Zoology, University of Otago, 340 Great King Street, Dunedin 9054, New Zealand

**Keywords:** DNA methylation, Zebrafish, Genome sequencing, RRBS, Brain, Liver

## Abstract

Zebrafish (*Danio rerio*) is a vertebrate model organism that is widely used for studying a plethora of biological questions, including developmental processes, effects of external cues on phenotype, and human disease modeling. DNA methylation is an important epigenetic mechanism that contributes to gene regulation, and is prevalent in all vertebrates. Reduced representation bisulfite sequencing (RRBS) is a cost-effective technique to generate genome-wide DNA methylation maps and has been used in mammalian genomes (e.g., human, mouse and rat) but not in zebrafish. High-resolution DNA methylation data in zebrafish are limited: increased availability of such data will enable us to model and better understand the roles, causes and consequences of changes in DNA methylation.

Here we present five high-resolution DNA methylation maps for wild-type zebrafish brain (two pooled male and two pooled female methylomes) and liver. These data were generated using the RRBS technique (includes 1.43 million CpG sites of zebrafish genome) on the Illumina HiSeq platform. Alignment to the reference genome was performed using the Zv9 genome assembly.

To our knowledge, these datasets are the only RRBS datasets and base-resolution DNA methylation data available at this time for zebrafish brain and liver. These datasets could serve as a resource for future studies to document the functional role of DNA methylation in zebrafish. In addition, these datasets could be used as controls while performing analysis on treated samples.

**Table:** 

Specifications	
Organism/cell line/tissue	*Danio rerio*
Sex	Male and female
Sequencer or array type	Illumina HiSeq2000
Data format	Raw and processed
Experimental factors	Tissue (wild type)
Experimental features	Genome-wide DNA methylation profiling of zebrafish male and female brain and zebrafish liver.
Consent	Level of consent allowed for reuse if applicable
Sample source location	Otago Zebrafish Facility, Dunedin, New Zealand

## Direct link to deposited data

The datasets supporting this article are available in the NCBI Gene Expression Omnibus (GEO) archive. Accession number for the Brain data is GSE59916. Link to the data: http://www.ncbi.nlm.nih.gov/geo/query/acc.cgi?acc=GSE59916.

Accession number for the Liver data is GSE59917. Link to the data: http://www.ncbi.nlm.nih.gov/geo/query/acc.cgi?acc=GSE59917

## Experimental design, data analysis and usage

### Original purpose

These datasets enabled us to describe the first RRBS study in zebrafish and compare the zebrafish methylome with that of mammalian genomes (e.g., human, mouse and rat) to highlight the technical and biological differences between these species [6]. To confirm that the high level of methylation we observed in the zebrafish reduced-representation (RR) genome is not confined to brain cells, we performed methylation sequencing of DNA from the liver. The liver dataset was also used as a reference to highlight differential methylation patterns between male and female brains (Chatterjee, A et al., in preparation).

### Sample description

For the brain methylomes, 12 male and 12 female brains were dissected and halved through the sagittal plane. Then two separate pools of male brains (referred as Male 1 and Male 2) and female brains (Female 1 and Female 2) were prepared, each consisting of six halved brains. The liver methylome was prepared from a pool of five male and five female livers harvested by dissection. DNA for RRBS library preparation was taken from these respective pooled samples (Table 1).

### Methylation sequencing

RRBS library preparation was constructed using a previously published protocol [7]. Four zebrafish brain libraries were sequenced using the Illumina HiSeq2000 platform (Illumina, San Diego, CA) in a single-ended (SE), 49 bp run (Beijing Genomics Institute, China). The liver library was sequenced (100-bp single ended reads) at New Zealand Genomics Limited (University of Otago, New Zealand). Sequence files were in obtained in FASTQ format.

### Quality assessment of sequence data and post-processing

Data quality was checked with the FastQC application (Babraham Institute, Cambridge, UK). The sequenced reads had a median Phred score of > 30 up to the last sequencing cycle for all brain methylome sequences. For the liver methylome samples, the quality decreased towards the end of sequenced reads, therefore the reads were hard-trimmed from 100 bp to 65 bp to improve data quality. The adaptor sequences from the reads were removed with the cleanadaptors program of the DMAP package as previously described [8], [22]. The brain methylome dataset (read length = 49 bp) contained negligible levels of adaptor sequences (evaluated with cleanadaptors and FastQC).

### Alignment to the reference genome

The sequenced reads were aligned against the zebrafish reference genome Zv9 using the bisulfite alignment program Bismark v0.6.4, or later, with a stringent criteria of one mismatch in the seed of 28 bp (default = 2) [13]. Bismark produces SAM files containing aligned reads with fields indicating the methylation status of CpG and other C nucleotides. The unique alignment efficiency ranged from 27.0% to 40.4% [6]. SAM files were converted to BAM files with the SAMtools [14] package to prepare data for submission to GEO (Gene Expression Omnibus) database. For example, the SAMTOOLS command used in Linux platform was: samtools view -bS ZFL_r1_adtr3pp.fastq_bismark.sam>ZFL_r1_adtr3pp.fastq_bismark.bam

### Availability and requirements

The sequencing data of zebrafish brain and liver is submitted to the NCBI GEO repository under two different accession numbers (Table 1). Both datasets consist of a metadata spreadsheet providing a summary of the project and files. As processed files, both datasets contain one .txt file per methylome describing the methylation status of each CpG site. These text files were generated using R package of methylKit [1]. The SAM files produced by Bismark were supplied as an input to methylKit and the CpG sites covered by at least 10 sequenced reads were retained to generate the text files. Mean coverage obtained on these CpG sites ranged from 21.4 to 77.25 between five methylomes [6]. Each CpG site was assigned a percentage methylation score. It is possible for individuals to use the raw SAM files (these can be converted from BAM files using SAMtools [14]) and generate these .txt files with different thresholds of CpG coverage if required. These .txt files enable easy access of the methylomes and the methylation status of any included CpG sites can be queried. In our submission, as raw files, BAM format files were provided comprising sequenced reads from four brain (Total size: 2.89 Gb) and one liver samples (size: 319.2 MB). These data files can be downloaded using File Transfer Protocol (FTP).

Project name: i) Genome-wide DNA methylation map of Zebrafish male and female brain and ii) Genome-wide DNA methylation map of Zebrafish liver

Operating system(s): Platform-independent, but UNIX/Linux preferred.

### Data requirements

After downloading, the data can be directly used for visualization. BAM files can be sorted and then imported in to Integrated Genome Viewer (IGV) for visualization of methylation data and this operation can be performed in a machine with 8 Gb RAM and 4 CPU cores. Differential methylation analyses can be performed within these samples or with other datasets, and will depend on the research question and study design.

## Discussion

Zebrafish is one of the most widely used model organisms in biological research, with many potential biomedical applications owing to the easy availability of hundreds of externally developing embryos. DNA methylation represents a stable epigenetic mechanism that is involved in gene regulation, and which has been implicated in human diseases, especially cancer [4]. Previous studies have suggested that the DNA methylation signature of the zebrafish genome is similar to that of mammalian genomes [11], making zebrafish an attractive model to study potential roles and mechanisms of altered DNA methylation in vertebrates.

Despite the importance of DNA methylation studies to the molecular understanding of development and biomedicine applications, the availability of high-resolution DNA methylation data for zebrafish is limited to date. Two recent studies provided whole genome bisulfite sequencing (WGBS) data for gametes and early stages of zebrafish development [12], [20]. However, in other published studies, either methylation data was generated using antibody pulldown techniques (e.g., MeDIP, which does not provide base-resolution information), or a limited number of CpG sites were investigated [2], [9], [16], [18]. Reduced representation bisulfite sequencing (RRBS) is a cost-efficient alternative to WGBS and has been shown to generate reproducible methylomes by several groups [5], [10], [17], [21]. RRBS has been widely used for genome-wide methylation profiling of human and mouse genomes, but has not been applied to zebrafish genome. Here we provide the first single-nucleotide resolution DNA methylome for the zebrafish brain and liver. We believe that the availability of these datasets will facilitate epigenetic research in this popular model organism.

The use of genome-scale approaches in zebrafish is on the increase, and will enable better understanding of biological and developmental processes commonly modeled using this animal. For example, global transcription initiation has been mapped at 12 stages of zebrafish development [19]. Furthermore, several studies have analyzed histone modifications and their predictive role in transcription (for example [15]). Whole genome methylation analysis, while still limited in scope, has been used to show that, following fertilization, the embryo methylome is adjusted to that of the sperm [12], [20], indicating that important biological information can be retrieved from the analysis of DNA methylation data. Of significance to our study, gene expression changes have been recently analyzed in the aging zebrafish brain in a comparison of male and female [3]. The addition of our RRBS datasets to those of emerging genome-wide studies in zebrafish should facilitate comparisons between studies, provide valuable correlative information, and accelerate the development of online hubs to enable future comparisons of zebrafish datasets.

## Figures and Tables

**Table 1:** Details of the zebrafish methylome datasets described in this DataNote.

Datasets	Tissue (pool)	Number of sequenced reads (million)	Read length (bp)	Uniquely mapped reads (million)	BAM files (megabyte)	Text file sizes (megabyte)	GEO accession number
Male1	Brain (n = 6)	24.48	49	8.01	757.2	16.8	GSE59916
Male2	Brain (n = 6)	24.49	49	7.93	754.5	15.9	GSE59916
Female1	Brain (n = 6)	24.49	49	6.61	649.0	12.0	GSE59916
Female2	Brain (n = 6)	24.49	49	7.57	725.2	16.0	GSE59916
ZF liver	Liver (n = 10)	9.0	100	3.64	319.2	8.60	GSE59917
